# Defining the interorgan communication network: systemic coordination of organismal cellular processes under homeostasis and localized stress

**DOI:** 10.3389/fcimb.2013.00082

**Published:** 2013-11-19

**Authors:** Ilia A. Droujinine, Norbert Perrimon

**Affiliations:** ^1^Department of Genetics, Harvard Medical SchoolBoston, MA, USA; ^2^Howard Hughes Medical InstituteBoston, MA, USA

**Keywords:** interorgan communication network, non-cell autonomous signaling, systemic disease, organismal homeostasis, systemic stress response

Following the acquisition of multicellularity, organisms with increasing levels of specialized cells, tissues, and organs emerged during evolution. To coordinate specialized organs, long-distance interorgan communication systems appeared. The central nervous system evolved to regulate many organ behaviors, using hormones or neurons. In addition, organs developed systems to directly communicate their states to one another. This is illustrated by the lack of nervous systems in plants and simple animals like sponges, which can perform complex systemic functions (Lough and Lucas, 2006; Srivastava et al., 2010).

Developmental or homeostatic events within cells or tissues have been extensively studied. For example, maintenance of the integrity of the *Drosophila* gut involves stem cell proliferation and differentiation, partially driven by local JAK/STAT, EGF, MAPK, and Wnt signaling (Panayidou and Apidianakis, 2013). Recently, it has become clear that individual organs themselves are also able to communicate their states. However, the nature of the interorgan signaling mechanisms remains largely a mystery.

Here, we review the emerging data supporting the existence of a vast interorgan communication network (ICN). The ICN is the network of peptides, proteins, and metabolites that act between organs to coordinate essential and specialized cellular processes under homeostasis and stress (Figure 1). We propose that studies in *Drosophila*, where, unlike in mammals, biochemical studies can be combined with genome-wide *in vivo* tissue-specific genetic screens, are poised to identify many ICN components. Characterization of the ICN will further understanding of systemic diseases such as cancer-associated muscle cachexia.

**Figure 1:**
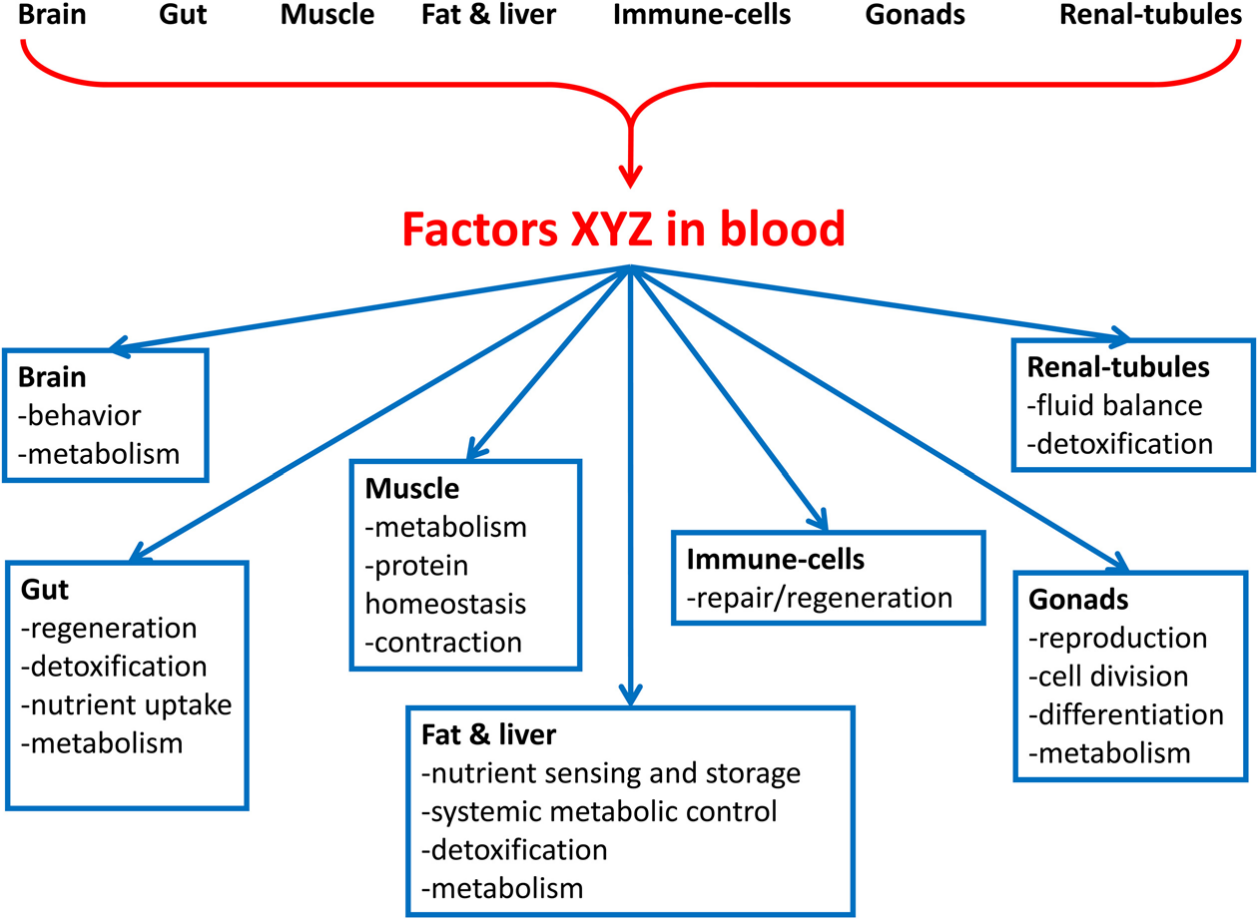
**Overview of the interorgan communication network (ICN). The ICN is the network of peptides, proteins, and metabolites that act between organs to coordinate organismal cellular processes under homeostasis and stress. Organs in the body secrete factors that act to influence the physiology of cells in distal organs. Processes that may be connected between organs include aging, protein homeostasis, nutrient uptake, metabolism, cell division, cell movement, detoxification, organelle biogenesis, and secretion of local and systemic signals. The signals may be nutrients, wastes, toxins, metabolites, nucleic acids, proteins, and peptides**.

## Function of the ICN: systemic integration of homeostasis

A limited number of studies in mammals, *C. elegans*, and *Drosophila* showed that perturbed tissues affect organismal growth and metabolism via largely unknown signals. The *Drosophila* fat-body (liver and adipose functional equivalent) responds to dietary signals by releasing factors affecting insulin secretion, growth, and metabolism (Britton and Edgar, 1998; Colombani et al., 2003; Géminard et al., 2009). For instance, in response to high dietary fat and sugar, the fat-body-derived leptin-like factor Unpaired-2 systemically controls release of insulin from insulin-producing cells in the brain (Rajan and Perrimon, 2012). Further, unknown nutrition-dependent signals control intestinal, neural, and germline stem cell division through local or systemic insulin signaling (LaFever and Drummond-Barbosa, 2005; Chell and Brand, 2010; O'Brien et al., 2011; Sousa-Nunes et al., 2011). Also, localized organ growth perturbations delay systemic development via inhibition of insulin signaling (DiAngelo et al., 2009), and insulin (Karpac et al., 2011) and ecdysteroid synthesis, partially through insulin-like Dilp8 (Colombani et al., 2012; Garelli et al., 2012).

In mammals, leptin is secreted by adipose tissue with nutritional surplus, controlling the neuroendocrine system (Zhang et al., 1994; Ahima et al., 1996). Also, exercise and muscle overexpression of PGC1-α increases the production of the secreted factor Irisin, a fragment of the transmembrane protein FNDC5, which stimulates metabolism and fat browning (Böstrom et al., 2012). Moreover, exercising muscle secretes interleukin-6 (Steensberg et al., 2000), possibly regulating systemic glucose and lipid metabolism by acting on muscle, liver, fat, intestinal L-cells, and pancreatic alpha-cells (Febbraio et al., 2004; Petersen et al., 2005; Ellingsgaard et al., 2011; Pedersen, 2011; Pedersen and Febbraio, 2012). Interestingly, liver or muscle autophagy controls whole-body glucose and fatty-acid metabolism, partially through FGF-21 (Kim et al., 2013). Finally, a number of gut-derived hormones including gastrin, ghrelin, cholecystokinin, glucagon-like peptide-1, and others affect insulin secretion, systemic fatty-acid metabolism, and feeding (Drucker, 2007). Strikingly, metabolic control is conserved, as leptin can rescue *Drosophila* Unpaired-2 deficiency, and both function through similar neuronal circuits (Vong et al., 2011; Rajan and Perrimon, 2012).

Intracellular pathways induce factors which regulate aging, stress resistance, and distal cellular functions. In *C. elegans*, germ-line absence extends life-span (Arantes-Oliveira et al., 2002) and causes systemic proteasomal activity increase, via unknown signals (Vilchez et al., 2012). In addition, tissue-specific induction of mitochondrial (Durieux et al., 2011), cytoplasmic (van Oosten-Hawle et al., 2013), and endoplasmic reticulum (ER; Taylor and Dillin, 2013) unfolded protein responses result in their systemic propagation, via poorly characterized factors. Neurotransmitter signaling partially mediates ER stress (Taylor and Dillin, 2013), but not heat-shock response propagation (van Oosten-Hawle et al., 2013). Moreover, systemic signaling to the brain causes behavioral avoidance of the stress-inducer (Melo and Ruvkun, 2012).

In *Drosophila*, gut, muscles, and fat-body are essential in stress resistance and aging. Gut infection or oxidative stress induces fat-body anti-microbial peptide secretion via unknown mechanisms (Foley and O'Farrell, 2003; Wu et al., 2012). Fat-body overexpression of FOXO transcription factor increases lifespan (Giannakou et al., 2004). Moreover, adult muscle-specific overexpression of FOXO prevents aging of other organs by decreasing accumulation of protein aggregates and increasing autophagy (Demontis and Perrimon, 2010). In addition, activation of muscle TOR or p38-MAPK signaling controls systemic aging and stress resistance (Vrailas-Mortimer et al., 2011). Also, muscle fatty-acid metabolism is essential for lifespan-increasing effects of dietary restriction (Katewa et al., 2012). Moreover, maintenance of gut homeostasis by stem-cell expression of PGC-1 or FOXO targets improves lifespan and metabolic homeostasis (Biteau et al., 2010; Rera et al., 2011).

Also, exposure of old mice to young blood results in restoration of muscle and liver regeneration, suggesting that systemic factors control aging (Conboy et al., 2005). For example, GDF-11 is a BMP ligand which slows myocardial aging through unknown mechanisms (Loffredo et al., 2013). Interestingly, TGF-β has been implicated in regulating reactive oxygen species production in the aorta, endothelial structure, blood-pressure, and cardiomyocyte function (Buday et al., 2010).

Systemic factors also control cell proliferation and tissue regeneration. In *Drosophila*, distal wounds control gut proliferative homeostasis via unknown mechanisms (Takeishi et al., 2013). Moreover, insulin regulates intestinal stem-cell proliferation (Amcheslavsky et al., 2009; Choi et al., 2011). In mammals, muscle from dystrophin-mutant mice may remotely alter wound healing (Straino et al., 2004). Also, liver-secreted betatrophin controls pancreatic beta-cell proliferation (Yi et al., 2013).

Unknown factors may also be controlled by reproduction. In insects, mating and fertilization induces numerous uncharacterized transcriptional changes in multiple organs (Rogers et al., 2008; Avila et al., 2011). In *Drosophila* females, mating increases mating receptivity, feeding, and egg-laying; changes movement; and decreases lifespan (Fowler and Partridge, 1988; Barnes et al., 2008; Avila et al., 2011). Some changes are associated with transfer of male accessory gland peptides (e.g., sex peptide) to females (Wigby and Chapman, 2005; Carvalho et al., 2006). Conversely, systemic factors may control reproduction. For instance, in *Drosophila*, insulin controls female germline stem cell proliferation (LaFever and Drummond-Barbosa, 2005). In *C. elegans*, oocyte and germline maintenance during aging is regulated by TGF-β and insulin via unknown relay signals (Luo et al., 2010).

In addition, systemic factors may regulate offspring fitness. In mice, paternal diet influences offspring metabolism (Carone et al., 2010; Ng et al., 2010). Moreover, the injury of fathers' and grandfathers' livers increases the regenerative capacity of their offspring's livers (Zeybel et al., 2012). Similarly, in *Drosophila*, tissue-specific stress causes heritable developmental alterations (Stern et al., 2012).

Finally, because alterations in its composition influence systemic physiology (e.g., metabolism; Claus et al., 2008), the microbiome is part of the ICN. For instance, obesity-induced changes in gut microbiome increase systemic deoxycholic acid that acts as a liver DNA-damaging and cancer-promoting agent (Yoshimoto et al., 2013).

In conclusion, there is growing evidence that many organismal functions mediate various aspects of interorgan communication through secreted factors. Understanding the roles of these factors, and how their activities are integrated to the organism's functions is the next big challenge. Further, as systematic screens have not been performed for such factors, it is likely that many additional ones remain to be identified.

## Structure of the ICN

Gene-expression analyses of organs have shown the existence of organ-to-organ coexpression networks that change in disease and aging, suggesting of unexplored interorgan processes and common responses of tissues to systemic factors (Keller et al., 2008; Dobrin et al., 2009; Huang et al., 2011). These analyses revealed that at least 40% of the interorgan features are not in single-tissue networks, and that the highly connected genes in the interorgan networks are poorly connected in the single-tissue networks (Dobrin et al., 2009).

What are the factors/nodes that connect the organs/hubs in the ICN? At their simplest and most evolutionary ancient form, signals may be nutrients, wastes, toxins, or metabolites. For instance, liver-produced beta-hydroxybutyrate inhibits histone deacetylases (Shimazu et al., 2013). Communication may also be in the form of circulating nucleic acids (e.g., miRNAs; Mitchell et al., 2008). Finally, proteins and peptides may be classical developmental regulators or novel. Intriguingly, “intracellular” proteins can be secreted outside the cell, as an isoform containing a signal sequence (e.g., PTEN-long; Hopkins et al., 2013), or through non-classical secretion (e.g., aP2; Cao et al., 2013)

An important feature that differentiates local tissue and developmental networks from the ICN, is the large distance over which signaling acts, meaning that concentration and specificity of the factors could be lower. To remedy this, a dense network of closely acting factors could exist, such that one factor acts on a neighboring tissue, which secretes a relay signal. Alternatively, signals may be carried along “molecular tracks” to their destination. These may be blood vessels or tissue regions containing “guidance factors”—putative weak affinity receptors to common structural features to groups of secreted factors. In addition, binding proteins (Mantovani et al., 2001) or proteases may be secreted to modulate local or systemic signaling. For example, *Drosophila* insulin-binding proteins ImpL2 (Honegger et al., 2008) or secreted decoy of insulin (Okamoto et al., 2013) bind to and inhibit insulin, locally or systemically. The mammalian ImpL2 homologs, insulin-like growth factor (IGF) binding proteins transport and regulate IGFs (Hwa et al., 1999; Honegger et al., 2008).

Factors may also be modified with fatty-acids, cholesterol, or glycans, regulating their stability, transport (Nusse, 2003; Linder and Deschenes, 2007; Moremen et al., 2012), and interaction with abundant and stable components including apolipoproteins (Panáková et al., 2005). These molecules can then deliver factors to target organs. For example, Hedgehog can be lipidated, interact with apoliproteins, and act distally (Palm et al., 2013). Finally, signaling can occur extracellularly through protease cascades (e.g., *Drosophila* spatzle-Toll; Morisato and Anderson, 1994) or phosphorylation (Yalak and Vogel, 2012).

## ICNs in human biology and disease

Elucidation of the ICN will be valuable for disease biology. Many disorders begin locally, and ultimately involve the entire organism by affecting behavior, cell recruitment, metabolism, proliferation, and activation (McCance and Huether, 2002). For example, muscle defects are associated with alterations in wound healing (Straino et al., 2004), regeneration, hepatocyte proliferation (Conboy et al., 2005), dyslipidemia, hypertension, type 2 diabetes, cardiovascular diseases, cancer, Alzheimer's and Parkinson's diseases (Pedersen, 2011). Moreover, cachexia, wound-healing, and hematopoiesis defects occur in cancer (Devereux et al., 1979; Egeblad et al., 2010).

Also, organ failure patients who receive organ function replacement therapy eventually succumb to disease, with systemic defects. For instance, kidney failure patients receiving kidney function replacement hemodialysis suffer from malnutrition and lung defects (McCance and Huether, 2002; Doi et al., 2011; White et al., 2011). This suggests that organs have essential functions beyond their “classic” roles, for example, by regulating distal organs through secreted factors. Importantly, blood-borne signals mediate critical systemic homeostatic adjustments from local perturbations, illustrated by control of systemic physiology by electrical cycling of paralyzed muscles in spinal-cord injured tetraplegic humans (Kjaer et al., 1996; Pedersen, 2011).

## Conclusions

Great strides are being made toward understanding intracellular and tissue homeostasis. The next step is to understand the structure, function, and components of the ICN. The main questions are the nature of the interorgan communication factors and their roles in maintaining whole-organism homeostasis. Also, how does the ICN change during development, aging, and disease? The current transcriptomic, proteomic, metabolomic, and genome-wide tissue-specific genetic manipulation technologies will allow answering these questions. Importantly, systematic *in vivo* identification of systemic factors is impractical in mammals. Thus, the ICN may be constructed for *Drosophila*, for which all of the above tools are available, and applied to mammals. Thus, “organ-sensing” RNAi screens can now be done, where genes are inactivated by tissue-specific RNAi, and function of another organ is assessed. Within the next decade, we expect a surge of interest to define the structure and function of the ICN.
